# Indents

**Published:** 1903-01-15

**Authors:** 


### INDENTS.

☞ **Brief Articles of Technical or Scientific Value will receive notice and comment. Contributions invited.**

**Perfectly Fitted Clasps.** Dr. G. W. Pitts, in the *Dental Review*, for June, describes his method of constructing clasps. First take an impression of the tooth for which you intend to construct clasp. Treat in usual way, and fill with investment compound for model. By adding a little whiting or prepared chalk to investment, one can acquire a great deal smoother model, a thing which is much desired at this point. About the tooth form burnish a very thin piece of platinum about the shape you intend to have clasp when completed, leaving it flush and extending well into the sulci. Wax securely, and fit approximately over it clasp metal thirty gauge. When it fits to your satisfaction run melted wax between platinum and clasp metal, allow to cool, and invest model platinum matrix and clasp metal. When investment has hardened, remove wax with hot water and heat case until all moisture has evaporated. To complete, flow twenty carat solder between platinum and clasp metal. Finish in the usual way.

**System and Order in the Dental Office.** It is beyond belief that men practicing dentistry should fail to realize the necessity for preserving order in the conduct of their work if they are to accomplish as much as they may. The very nature of the occupation is such that it demands one or two things—either system or chaos.

There are so many instruments and appliances, each for its own particular purpose, that to keep them promiscuously strewn about means confusion, and worse than this it means a most serious loss of time.

Would you go into any ordinary machine shop and expect to find the tools scattered about in utter disregard of where they properly belonged? And if you did, would you expect to have a piece of work turned out of that shop with any degree of accuracy or promptness? Would you have confidence that anything a slovenly and disorderly machinist did would be done in the most perfect manner?

And yet to go into many of our dental offices, where more tools are used than in the average machine shop, one would think that an earthquake had recently gone along and shaken things together. Think of the waste of time that would aggregate from a computation in all the dental offices were untidy methods prevail. And then think of the impression on people of discriminating taste who take a seat in a dental chair where the floor is littered with waste cotton and other refuse, and where the operating case looks as if it had just passed through an attack of the blind staggers. Does such a condition of affairs tend to establish and maintain a liberal patronage?

It would not be so bad if the lack of system was confined to the older men, who grew up in the profession at a time when professional requirements were not so exacting as they are to-day, but for the younger men to exhibit carelessness in this respect has a tendency to lower the general tone of the profession, besides being a perpetual handicap to the individual himself.

Be tidy, neat and systematic, and you will not only create a good impression on others, but will advance immeasurably in your own self-esteem.—Editorial in *Dental Review*.

**Fatalities.** A young woman at Camden, N. J., is dying from lockjaw following the extraction of a second molar.—A girl in Chicago recently tried to relieve toothache by putting a quantity of carbolic acid in the cavity. She accidentally swallowed considerable of the acid, and for a long time her life was despaired of, but she will recover.—A man at Salina, Kan., took gas for the extraction of a tooth, and for several hours afterwards was so dizzy that he could not walk.—A young woman at Blairsburg, Ia., almost bled to death last month from the extraction of a tooth, but is slowly recovering.—A woman at Wilkesbarre, Pa., recently had several teeth filled and crowned. A few days later her teeth began to ache, her gums became inflamed, her limbs swelled, and she had a peculiar metallic taste in her mouth. The physicians in the hospital to which she was taken diagnosed her case as one of metallic poisoning, and discovered that the crowns were made of brass and not gold. The woman died a few days later and the coroner is investigating. —A fourteen-year-old boy at Rose Point, Pa., died July 28th in a dentist's chair while having some teeth extracted. A physician administered the anesthetic.—July 14th a woman at Massillon, O., died under the influence of chloroform given prior to tooth extraction.—September 12th, a woman at Taylorville, Ill., died under the influence of chloroform while having a tooth extracted.—August 24th, a man at Sandusky, O., had a tooth extracted and almost died from hemorrhage.—August 28th, a boy at Denver, Colo., had a tooth extracted and it was a week before the flow of blood could be stopped. He came from a family of "bleeders."—August 29th, a three-year old child at Newport, Pa., died from blood-poisoning, resulting from the extraction of a tooth.—September 11th, a woman at Niles, O., died under chloroform given for tooth extraction.—August 28th, a boy at Toledo, O., had some teeth extracted by a street fakir and lockjaw set in. His recovery, however, is expected.—September 11th, a woman in Chicago died under gas given prior to tooth extraction.—August 31st, a young woman in Brooklyn had a tooth extracted, and the hemorrhage was so great that for sometime her life was despaired of.—September 1st, a woman in Chicago died under chloroform given prior to tooth extraction.—September 11th, a young woman at New Orleans died under chloroform. Three administrations were made, as she had several teeth to be taken out.—*Digest.*

**Tooth Brushes.** Nearly all brushes are made from bristles taken from the wild hogs of Russia or China. The handles are common beef bones. They are made mostly in Japan, France, England and Germany, and by one firm in the United States. Probably English brushes are the best made and worst shaped. The French are next in quality, but far ahead in form. Germany and Japan are generally imitators. Some of the most expensive English and French, and all American brushes, are made in factories under more or less sanitary conditions, but the cheaper grades, including all German and Japanese brushes, are made in the huts of the peasants, where cattle, swine, dogs, fowls and humans are herded in common. The bristles and bone are given out by the dealer and taken into the country, and there assembled by the aged and young children and diseased persons, the stronger members of the family working at more remunerative employment.

These cheap brushes are often made in the most unsanitary and wretched surroundings imaginable, and it is a significant fact that after being made they are seldom sterilized before using. The English brushes are generally very much too large to be efficient. The French are better shaped, but are apt to be too long of head, making much waste to the brush, and are too long of bristle.

A wide brush is not advisable because it limits the movement possible longitudinally to the tooth. Long bristles are not best because they bend when the brush is thrust back between cheek and teeth and stay bent till the brush is withdrawn, thus missing the interproximal spaces so much in need of cleaning. Soft bristles become softer when wet and utterly fail to enter the spaces at all. If the surface of the bristles is concaved longitudinally to fit the labial curve of the teeth, then when the brush is reversed and used on the lingual surfaces, only the ends of the brush engage the teeth; hence, more teeth are missed than cleaned, and the user is deceived into thinking he has cleaned his teeth because he has brushed them.

Studying the brush over and what is required of it, it would seem that the brush best adapted to use in the human mouth should have a short, narrow head with short, rather stiff bristles, trimmed straight longitudinally and convex latitudinally, that each line of bristles may come successively into use as the brush is rotated. —L. H. Arnold, in *Review*.

